# Integration of Traditional Healers in Human African Trypanosomiasis Case Finding in Central Africa: A Quasi-Experimental Study

**DOI:** 10.3390/tropicalmed5040172

**Published:** 2020-11-17

**Authors:** Sylvie Kwedi Nolna, Rodrigue Ntonè, Nicole Fouda Mbarga, Severin Mbainda, Willy Mutangala, Bernard Boua, Miriam Niba, Aline Okoko

**Affiliations:** 1 Faculty of Medicine and Biomedical Sciences, University of Yaoundé 1, Yaoundé, Cameroon; 2 Capacity for Leadership Excellence and Research (CLEAR), Yaoundé, Cameroon; miriam20niba@gmail.com; 3 Epicentre, Medecins Sans Frontières, Yaoundé, Cameroon; Rodrigue.NTONE@epicentre.msf.org (R.N.); tmbarg200@gmail.com (N.F.M.); 4 Ministry of Health, National HAT Program, N’Djamena, Chad; soulcvr1@yahoo.fr; 5 Ministry of Health, National HAT Program, Kinshasa, Democratic Republic of Congo; willymutangala@yahoo.fr; 6 Ministry of Health, National HAT Program, Bangui, Central African Republic; Bernard.boua@gmail.com; 7 Organisation de Coordination pour la lutte contre les Endemies en Afrique Centrale (OCEAC), Yaoundé, Cameroon; Aline.okoko@gmail.com

**Keywords:** Trypanosomiasis, sleeping sickness, case finding, screening, traditional healer, case finding, screening, traditional healer

## Abstract

Background: Based on the premise that Africans in rural areas seek health care from traditional healers, this study investigated a collaborative model between traditional healers and the national Human African Trypanosomiasis (HAT) programs across seven endemic foci in seven central African countries by measuring the model’s contribution to HAT case finding. Method: Traditional healers were recruited and trained by health professionals to identify HAT suspects based on its basics signs and symptoms and to refer them to the National Sleeping Sickness Control Program (NSSCP) for testing and confirmatory diagnosis. Results: 35 traditional healers were recruited and trained, 28 finally participated in this study (80%) and referred 278 HAT suspects, of which 20 (7.19%) were CATT positive for the disease. Most cases originated from Bandundu (45%) in the Democratic Republic of Congo and from Ngabe (35%) in Congo. Twelve (4.32%) patients had confirmatory diagnosis. Although a statistically significant difference was not shown in terms of case finding (*p* = 0.56), traditional healers were able to refer confirmed HAT cases that were ultimately cared for by NCSSPs. Conclusion: Integrating traditional healers in the control program of HAT will likely enhance the detection of cases, thereby, eventually contributing to the elimination of HAT in the most affected communities.

## 1. Introduction

Human African Trypanosomiasis (HAT) or Sleeping Sickness is a vector-borne parasitic disease, endemic in 36 African countries with 60 million individuals at risk of getting the disease [1]. HAT appears to have a 100% fatality rate [2]. It is caused by *Trypanosoma brucei gambiense*, which is a chronic form of the disease present in western and central Africa, and by *Trypanosoma brucei rhodesiense*, which is responsible for acute disease in eastern and southern Africa. Central African countries such as Cameroon, Central African Republic, Congo, Gabon, Equatorial Guinea, Democratic Republic of Congo (DRC) and Chad account of 77% of all new *T. b. gambiense* cases reported globally between 2000 and 2016 [3]. The World Health Organization (WHO) aims at eliminating gambiense human African trypanosomiasis (g-HAT) as a public health problem by 2020 [4]. So far, the global incidence of HAT has reduced by 95% since the beginning of this century. However, case finding remains a major challenge faced by National Sleeping Sickness Control Programs (NSSCPs) [5]. In Gabon, according to the activity report of the National Program for the Control of Parasitic Diseases in 2015, the coverage rate of households in active screening was 33% and 34% in the Republic of Congo. In DRC, the country with the largest population at risk for HAT, the inability of active case-finding surveys to cover all transmission areas has been noted [6]. Elimination of g-HAT will require integrated solutions that take into consideration the environment and the culture of these affected communities. In Africa, traditional healers are recognized as key players in health care due to their cultural-focused approach in health promotion and disease prevention [7] and 85% of the population in Sub-Saharan African countries seek traditional medical services [8]. In South Africa for instance, traditional healers have been found to play a vital role in the primary health care of rural populations in Limpopo Province [4].

Unfortunately, traditional healers are seldom involved in HAT detection and control strategies. NSSCPs mainly carry out active and passive case finding to reduce transmission of HAT in affected areas [9]. Hence, this study sought to evaluate the feasibility of establishing a collaboration between the NSSCPs and traditional healers for enhanced HAT case finding in central African foci. This strategy does not only make use of a community-accepted health care solution, but it taps into a novel collaborative model for rampant public health issues.

## 2. Methods

### 2.1. Study Type

A prospective quasi experimental study design (pre and post) was used to assess the feasibility of integrating traditional healers in HAT case finding in Central Africa. The Transparent Reporting of Evaluations with Nonrandomized Designs (TREND) statement checklist served as a framework for reporting this study [10].

### 2.2. Setting

The study was conducted in seven central African countries: Cameroon, Central African Republic (CAR), Chad, Democratic Republic of the Congo, Equatorial Guinea, Gabon and Republic of Congo from May to November 2016. These countries were selected based on 2012 estimates and mapping of the population at risk of HAT in Africa [6].

At the time of study implementation, the Democratic Republic of Congo counted 20 foci, while the other six countries had between four and six foci each [3,11].

In all seven intervention countries, HAT control activities are coordinated by NSSCP, as part of the Ministry of Health. They use various disease control strategies such as screening, diagnosis, treatment and vector control to reduce the disease reservoir.

### 2.3. Study Population and Sampling

Convenience sampling was used to select traditional healers for participation in the study. The first five traditional healers (18 years and older) identified in the affected communities were approached and invited to participate in the study. The healers who consented to partake in the study were trained on the collaborative model. In each country, the NSSCP nominated one to three of their team members (healthcare professionals) to train the traditional healers in study procedures. All patients aged 18 years and above who visited participating traditional healers were systematically screened based on seven main symptoms: fever, swollen lymph nodes, arthralgia (joint pain), irritability, pruritus (itchy skin), severe headache and extreme fatigue. Inclusion in the study was proposed to all patients with at least one symptom at disease screening. Suspect cases were only referred to NSCCP centers for diagnosis after providing written informed consent.

Although the limitations of convenience sampling are fully acknowledged, this sampling method was applied as it is efficient and simpler to implement in a context where traditional healers and not registered by any authority and thereby not inventoried.

### 2.4. Study Procedure

The traditional healers were trained by the NSSCPs on the basics of etiology, pathophysiology, diagnosis of HAT, and most importantly, on the clinical signs and symptoms of HAT (fever, swollen lymph nodes, arthralgia (joint pain), irritability, pruritus (itchy skin), severe headache and extreme fatigue), as well as community approaches for HAT control. The training was aimed at providing the traditional healers with the required knowledge to identify suspected HAT cases for referral to NSSCPs for diagnosis of HAT. The card agglutination test for trypanosomiasis (CATT) was performed as a screening test on suspected cases at the NSSCP center followed by parasitological examination of body fluids (blood or cerebrospinal fluid) for confirmation.

### 2.5. Data Collection Tools

Each suspected HAT case identified by traditional healers received a “pink card” that was collected upon the patient’s arrival at the referred NSSCP center. This “pink card” distinguished study participants from the rest of their patients at the NSSCP center. The HAT diagnostic test was performed at the NSSCP center and results were reported monthly to investigators. All the pink cards collected by NSSCP centers were counted and compared to the number of pink cards left with the traditional healers in order to identify the referred suspects who did not arrive at the NSSCP centers.

## 3. Data Analysis

The overall effect of the intervention strategy was measured by comparing the CATT results for the total number of suspected cases received in the NSSCP centers of the selected foci in the 6 months before and after the intervention using Microsoft Excel. The Chi square test was used to evaluate the proportion change in the number of reported cases in the 6 months before and 6 months after the training.

## 4. Ethical Considerations

This study involved several countries in Central Africa and was evaluated by the Ethics Committee for Research and Health in Central Africa (CERSAC) who provided ethical clearance (AVIS No. 003/CERSAC/BE/2016). Administrative authorizations were obtained from Ministries of Health of each participating country prior to implementation of the study. A written informed consent was obtained from study participants prior to study inclusion as prescribed by the International Council on Harmonization guidelines on Good Clinical practice.

## 5. Results

### 5.1. General Characteristics of Traditional Healers and Study Participants

This study was conducted in seven countries of Central Africa across seven foci (Table 1). Across all study sites, a total of 35 traditional healers were recruited and trained by 11 NSSCP health professionals (Table 1). Out of 35 traditional healers trained, 28 (80%) participated in the study and conducted study activities as planned. CAR had the lowest rate (20%) of non-participation of traditional healers, which may be due the political unrest in that country.

The 278 participants with “pink cards” found at NSSCP centers comprised 149 women and 129 men. Their ages ranged between 18 to 80 years with an average age of 32.82 ± 14.63 years.

### 5.2. Diagnostic Test Results of HAT

Table 2 shows that, of the 278 participants referred to NSSCP centers for diagnosis, 20 had a positive CATT test resulting in a positivity rate of 7.19%. The Bandundu focus in the Democratic Republic of Congo had 9 out of 20 CATT positive patients (45%), followed by the Ngabe focus in Congo with 7 CATT+ cases (35%). Out of the 20 CATT+ cases, 12 had a confirmatory diagnosis for HAT via microscopic examination of blood samples, thus yielding a prevalence of 4.32% confirmed HAT cases in our study population.

### 5.3. Effect of the Intervention

As seen in Table 3, the total number of positive cases detected after the training increased from 22 in the 6 months before the intervention to 33 in the same interval of time after the intervention. The prevalence increased from 1.87% to 2.21% before and after the training respectively. Pearson’s chi squared test revealed no statistical significance in the correlation between the suspected and confirmed cases before and after the intervention (*p* = 0.58).

## 6. Discussion

This study sought to evaluate the feasibility of establishing a collaboration between the NSSCPs and traditional healers as well as to assess the effect of this collaboration on HAT case finding in central African countries. Although statistical significance could not be demonstrated for the difference made by the intervention, the study was able to detect a CATT positivity rate of 7.19% with a prevalence of 4.32% of confirmed HAT cases in the study population. Without this study intervention, these patients diagnosed and treated may not have been found as traditional healers are not in the habit of referring suspected HAT cases to the NSSCPs. 

HAT elimination is unlikely to be achieved without improved case detection and management. Involving the community in the identification of suspected cases will not only cast a wider net but it will also lead to enhanced case finding strategies. Traditional healers have been shown to be highly motivated to collaborate with conventional medicine and public health officials in control efforts of several diseases [12,13,14].

The 4.31% prevalence of HAT reported during the 6 months intervention period of this study affirms that traditional healers provide healthcare services to affected individuals, confirming the healers’ potential contribution to the improvement of HAT case detection in Africa. Since these African indigenous health care providers are known to treat HAT cases with herbal medicine with trypanocidal effects [15,16], they may also be likely to detect HAT amongst patients in their facilities.

In a previous study aimed at evaluating the impact of integrating passive screening in primary health care services in three health districts of the Democratic Republic of Congo, 2.2% to 3% of CATT+ cases were found with a confirmation rate of 45.6% [9], which is statistically similar (p < 0.05) to the 7.2% of CATT+ and 60% confirmatory diagnosis observed in this study. Such data supports the integration of traditional healers as an asset to the fight against HAT in affected communities. Their involvement was also shown to be cost effective in the context of HAT surveillance through passive screening [17].

Although a statistically significant difference was not shown in terms of case finding (*p* = 0.56), traditional healers were able to refer confirmed HAT cases that were ultimately cared for by NCSSPs. Palmer et al. argued that HAT case finding should incorporate “expert and non-expert forms of diagnostic practice” [18] leading to conclude that working with traditional healers will only enlarge the pool of individuals capable of suspecting this disease in the most affected communities.

Collaborations with traditional healers have been shown to improve healthcare services and outcomes in other disease areas. Semenya reported that involvement of traditional healers led to optimization of Human Immunodeficiency Virus testing services in South Africa [19]. Similarly, training and engagement of traditional healers in active tuberculosis case detection has been proven effective in Burkina Faso [20], Ethiopia and Tanzania [21]. Seeking care from traditional healers is a widely accepted health-care solution in Sub-Saharan Africa [8]. This practice should add value to the improvement of public health in African communities. Thus, this study highlights the need for capacity building of traditional healers for the improvement of disease surveillance and control.

Although this study contributes to filling a research gap, it is not without limitations. The convenience sampling method was used to select traditional healers across all seven sites, which affects the generalizability of our findings.

Collaborating with traditional healers in identifying HAT suspect cases is an asset for national control programs of this disease. Future research should aim at assessing the impact of integration of active and passive HAT control activities involving traditional healers. Particularly qualitative research on the community’s ability to improve case finding will provide multidimensional insights for this disease’s elimination.

## Figures and Tables

**Table 1:** Participation of Traditional Healers.

Country	Focus	Health Area	Traditional Healers Trained (%)	Traditional Healers Involved in the Study (%Participation)	Health Personnel in Each Focus
Cameroon	Campo	Campo	5	5 (100)	1
Congo	Ngabe	Ngabe	5	5 (100)	1
Gabon	Como Kango	Commune Kango	5	2 (40)	2
Equatorial Guinea	Mbini	Mbini	5	5 (100)	2
Central African Republic	Nola	Nola	5	1 (20)	1
Democratic Republic of Congo	Badundu	Nkara	5	5 (100)	1
Chad	Mandoul	Mandoul	5	5 (100)	3
Total			35	28 (80)	11

Data are percentages calculated based on total number of traditional healers trained in each focus.

**Table 2:** Diagnosis Test Results of HAT Suspects.

Country	Focus	Number of Suspected Cases Referred	Number of CATT- Cases	Number of CATT+ Cases (%)	Number of Microscopic Confirmed Cases (%)
Cameroon	Campo	22	21	1 (0.36)	1(0.36)
Congo	Ngabe	81	74	7 (2.52)	4 (1.44)
Gabon	Como Kango	6	5	1 (0.36)	0 (0)
Equatorial Guinea	Mbini	8	7	1 (0.36)	1(0.36)
Central African Republic	Nola	13	12	1 (0.36)	1(0.36)
Democratic Republic of Congo	Bandundu	73	64	9 (3.24)	5(1.80)
Chad	Mandoul	75	75	0 (0)	0(0)
Total		278	258	20 (7.19)	12 (4.32)

Data are percentages calculated based on total number of HAT suspected cases.

**Table 3:** Comparison of the Number of HAT Cases Before and After 6 Months of Intervention.

	Total Number of Suspected Cases	Number of Negative Cases	Number of Confirmed Cases	Prevalence (%)	*p*-Value
Before the intervention	1177	1155	22	1.87	0.580
After the intervention	1405	1374	31	2.21	
